# Supplementary material for: Patterns of Intron Gain and Loss in Fungi
Source: PLoS Biol. 2004 Nov 30;2(12):e422. doi: 10.1371/journal.pbio.0020422 (PMC532390; doi:10.1371/journal.pbio.0020422)
Supplement: Table S1 — Also available at http://genes.mit.edu/NielsenEtAl/. (4.3 MB ZIP). [file pbio.0020422.st001.zip › NielsenEtAl/html/502.html]

AN0346.1.NCU01916.1.MG02974.1.FG08931.1


```
 CLUSTAL W (1.82) Multiple Sequence Alignments - Introns Inserted


Sequence 1: NCU01916.1	384 aa
Sequence 2: FG08931.1	385 aa
Sequence 3: MG02974.1	388 aa
Sequence 4: AN0346.1	351 aa
Alignment Length: 403 aa
Number Identitical Residues: 139 aa
Alignment Score (without introns) 7549


MG02974.1 	MMDSPQATATLVKSYVS------EDRVDRPPLAQSREIGGAVPIG-------RPEGAQAL
NCU01916.1	-MESLPATRRVPVPSDQ------HYKGDHIPLVVSRETGDIVPESHQADPLVRPDKIEAT
FG08931.1 	-MSHHQTSSQIMTRDDEKPFNHTHVPIDHAALIQSRETGDVLPD----DTPIQGN-VKAL
AN0346.1  	-MQAMRNDS------------------KDAALNAAVPEGPISPP-------------QVQ
          	 *.                        .  .*  :   *   *              :. 

MG02974.1 	NFAKSWSHFVAGG2IGGMTAAALTAPLDVLKTRLQSDFYQQQIRQRNVAVQSFSGLTGPF
NCU01916.1	PFAKSWAHFVAGG2IGGMTAAALTAPLDVLKTRLQSDFYQAQLRASRQSHVGVP--LNPI
FG08931.1 	PFAKSWVHMFAGG2VGGMTAAAITAPLDVLKTRLQSDFYQAQIRAQREAQVQTLGRLNPA
AN0346.1  	QTNSSWTHLVAGA2AGGMATALLTSPLDVLRTRLQSDYYRSQLKS---TTTSSHARFSLA
          	   .** *:.**.  ***::* :*:*****:******:*: *::    :     .  .  

MG02974.1 	RMMHFHLGETFAILGSVYKQEGPRALFKGLGPNLVGVIPARSINFFTYGNMKQLLSGHFN
NCU01916.1	RAAWYHFSETGQILSAVYRQEGPRALFKGLGPNLVGVVPARSINFFTYGNGKRLIAKYFN
FG08931.1 	RSALYHLNDTLQILSSVYKNEGWRALFKGLGPTTVGVVPARAINFYVYGNGKRLISEHFN
AN0346.1  	RSFIQHFSETFEILFSIHRVEGWRSLFRGLGPSLTGVVPATAIKFYAYGNCKRLYPEVFG
          	*    *:.:*  ** :::: ** *:**:****. .**:** :*:*:.*** *:* .  *.

MG02974.1 	GGIEADWINFVSAMTAGVVTSTATNPIWLIKTRLQLDKSTAAR--SGTGRQYRNSFDCLK
NCU01916.1	NGQEGTWVHLSAGVLAGIVTSTATNPIWMVKTRLQLDKNVAAESGGVTRRQYQNSYDCIR
FG08931.1 	NGVEAPWVHLSAGVAAGVTTSTATNPIWMIKTRLQLDKNVAA--GGAQMRKYRNSYDCIR
AN0346.1  	LDRDATSTHALSAATAGVVTGTATNPIWLVKTRLQLDRTHVNSDGTTRPPQYRNSYDCVK
          	 . :.   :  :.  **:.*.*******::*******:. .  ..     :*:**:**::

MG02974.1 	QVIRNEGVRGMYKGMSASYLGVTESTLHWVLYEQMKAALRRREEELVLSGRPKTTWDTVV
NCU01916.1	QILRNEGLRGLYKGMSASYLGVAESTLQWVLYERMKTALALREEKIVQSGRQKTWWDHAV
FG08931.1 	QILRDEGIRSLYRGMSASYLGVVESTMQWMLYEQMKVSLARRHNEIVRSGREKTWWDKTV
AN0346.1  	QVIRQEGIKGLYRGLAASYLGVIETTLHLASYERIKVAVARHYE-----RKGKTQSGEVT
          	*::*:**::.:*:*::****** *:*::   **::*.::  : :      : **  . ..

MG02974.1 	DYTGKVVAAGTAKLLAAVPTYPHE~VARTRLRQAPMADGRPKYTGLVQCLRTVWIEEGAA
NCU01916.1	NWTGNAGAAGGAKLVAAVLTYPHE~VARTRLRQAPMDGGKPKYTGLIQCFKLVFKEEGMA
FG08931.1 	DWTGKGFAAGSAKLVAAVIAYPHE0VARTRLRQAPMNNGLPKYTGLVQCFKLVWVEEGLM
AN0346.1  	QGLILSGSAAVSKLIAVLIAYPHE0VLRTRLRQAPMADGRQKYTGVLQCLRLMVKEEGFV
          	:      :*. :**:*.: :**** * ********* .*  ****::**:: :  ***  

MG02974.1 	GLWGGLTPHLLRTVPSAAIMFGMYEGILLLLNEPEAPKLDDQKAR
NCU01916.1	GLYGGMTPHMLRTVPSAAIMFGMYEAILRLLGTPS----------
FG08931.1 	GLYGGLTPHLMRTVPSAAIMFAMYEGILRLFHTPA----------
AN0346.1  	ALYGGLTAHMIRTVPSAAITLGTYELVLKLLSA------------
          	.*:**:*.*::******** :. ** :* *:
```
